# Open‐Source System Suitability: Mass Spectrometry Query Language Lab (MassQLab)

**DOI:** 10.1002/rcm.10132

**Published:** 2025-09-02

**Authors:** Heather L. Winter, Dylan Johnson, Alan K. Jarmusch

**Affiliations:** ^1^ Metabolomics Core Facility, Immunity, Inflammation, and Disease Laboratory, Division of Intramural Research National Institute of Environmental Health Sciences, National Institutes of Health, Research Triangle Park Durham North Carolina USA; ^2^ Integrative Bioinformatics, National Institute of Environmental Health Sciences National Institutes of Health, Research Triangle Park Durham North Carolina USA

**Keywords:** computational mass spectrometry, data science, lipidomics, metabolomics, quality assessment, quality control

## Abstract

**Rationale:**

Reproducible analytical instrumentation system performance is critical for mass spectrometry, particularly metabolomics, aptly named system suitability testing. We identified a need based on literature reports that stated only 2% of papers performed system suitability testing.

**Methods:**

We report MassQLab, built upon open‐source, vendor‐agnostic software called the mass spectrometry query language (MassQL). MassQL, implemented in MassQLab, provides freedom for researchers to choose their analyte/s, mass spectrometry system (including liquid chromatography—mass spectrometry), and metrics of performance.

**Results:**

In this report, we describe the use of MassQLab, demonstrate the construction of the required MassQL query, common metrics of performance (i.e., extracted ion chromatograms), uncommon metrics (i.e., MS/MS product ion spectra), and discuss insights gained about performance—including issues requiring correction prior to sample analysis.

**Conclusions:**

MassQLab is a flexible solution for system suitability testing for mass spectrometry‐based analytical measurements. Deficits in analytical performance, while unavoidable and rare, were noted prior to data collection and corrected. The open‐source and adaptable nature of MassQLab will empower researchers and lead to improved implementation of system suitability testing.

## 1 Introduction

The quality of mass spectrometry (MS) data is critical and should be assessed at different stages of the research process, such as sample processing, data acquisition, and data analysis. Differences in acquisition performance can be introduced from environmental conditions (e.g., temperature), calibration, and system condition (cleanliness). The differences, if left unaddressed, can result in the incorrect interpretation of data, such as inappropriately reporting amounts or misidentification of analytes.

System suitability testing (SST) aims to assess the performance of data acquisition systems prior to data collection [1]. Multiple evaluation options exist, including manual data inspection or use of software. Such evaluations may be limited to specific aspects of the overall measurement, such as MS mass accuracy or LC retention time, or complete systems (LC–MS) [2, 3, 4, 5]. The variety of published approaches mirrors the numerous MS and hyphenated MS system configurations and multiple acquisition strategies (e.g., data‐dependent acquisition [DDA]). The conceptualization of system suitability is thorough and mature; yet, the execution remains limited. Broeckling et al. reported that only 2% of 109 surveyed untargeted metabolomics papers used system suitability samples [6]. The authors believe that one factor is the lack of easy to implement software (with minimal coding experience required) that is universally applicable (different instrument vendors).

A new language developed for MS, dubbed the Mass Spectrometry Query Language (MassQL) [7], has been recently reported. The open‐source, flexible, and vendor‐agnostic attributes of MassQL make it well suited for use in system suitability assessment. Further, MassQL can be used in common coding languages such as R and Python. One prerequisite is that vendor‐specific file formats must be converted into an open‐source MS file format (i.e., .mzML). We sought to take advantage of the attributes of MassQL and implemented them into a package called MassQLab. In this report, we demonstrate the implementation of MassQL via MassQLab for SST using a mixture of six deuterated acylcarnitine standards on an ultrahigh performance LC–high‐resolution mass spectrometer (UHPLC‐HRMS).

## 2 Materials and Methods

### 2.1 Materials and SST Mixture

Chemical standards were purchased from Cayman Chemical to create the quality control (QC) mixture: acetyl‐L‐carnitine‐d_3_ (26564), propionyl‐L‐carnitine‐d_3_ (26579), valeryl‐L‐carnitine‐d_3_ (27871), octanoyl‐L‐carnitine‐d_3_ (26577), lauroyl‐L‐carnitine‐d_3_ (26571), and oleoyl‐L‐carnitine‐d_3_ (26578). Acetonitrile (Optima LC–MS grade, Fisher Chemical), methanol (Optima LC–MS grade, Fisher Chemical), water (HPLC‐grade, Fisher Chemical), and acetic acid (LiChropur HPLC‐grade, Sigma‐Aldrich) were used.

The SST mixture was prepared to a final concentration of 1 μg/mL via the addition of each deuterated standard into 100 mL volumetric glassware and dilution to the mark. The solution was mixed via inversion thoroughly; 1 mL aliquots were transferred to 2 mL autosampler vials (12 × 32 mm height vial, 12 mm screw cap, and PTFE/silicone septa, Agilent) and dried via centrifugal evaporation (Genevac EZ‐2 Plus, SP Scientific). Vials containing dried SST mixture were capped and stored at −80°C. Dried SST mixture was resuspended via the addition of 1 mL of 2% acetonitrile—98% water *v/v* and vortexed for ~3 s.

### 2.2 Mass Spectrometry Method

Samples were analyzed using an ultrahigh performance liquid chromatograph (Vanquish Horizon, Thermo Scientific) coupled to a high‐resolution mass spectrometer (Orbitrap Fusion or Fusion Lumos, Thermo Scientific). LC–MS and LC–MS/MS data were acquired on the SST mixture in positive ionization mode. Prior to measurement, the mass spectrometer was calibrated using FlexMix (Thermo Scientific) following manufacturer directions. Chromatographic separation was carried out on an F5 analytical column (2.1 × 100 mm, 100 Å, 2.6 μm, Phenomenex) with a corresponding guard cartridge. The column was maintained at 30°C during separation with a solvent preheater. Gradient elution was performed after an initial period of isocratic elution using water with 0.1% acetic acid *v/v* (A) and acetonitrile with 0.1% acetic acid *v/v* (B). Separation was performed as follows: 0% B from 0 to 2.0 min, 0% to 100% B from 2.0 to 10.5 min, 100% B from 10.5 to 12.0 min, 100% to 0% B from 12.0 to 13.0 min, and 0% B from 13.0 to 20.0 min. The flow rate was 0.5‐mL min^−1^. Ionization was performed via heated electrospray ionization (NG Ion Max, Thermo Scientific). The source parameters were as follows: spray voltage of +4000 V, sheath gas of 50 arbitrary units (arb), auxiliary gas of 10 arb, sweep gas of 1 arb, ion transfer tube at 325°C, vaporizer at 350°C. MS data were acquired at 120000 resolution from *m/z* 100 to 1000 with an RF lens of 60% and maximum injection time of 50 ms. MS/MS data were acquired at 30000 resolution using an isolation width of 1.5 (*m/z*) using collision‐induced dissociation (CID) and higher energy collision‐induced dissociation (HCD). MS/MS spectra were obtained using 10, 20, 30, 40, 50, and 60 normalized collision energy sequentially with HCD and then CID (q = 0.25, activation = 10 ms).

### 2.3 Data Processing With MassQLab

Raw data files (.raw) were converted to mzML format using MSConvert (https://proteowizard.sourceforge.io/) with default settings. Data are available on MassIVE (massive.ucsd.edu), accession number MSV000098825. A Jupyter notebook implementation of MassQL was developed in‐house to process data in mzML format (MassQLab). MassQLab is a Python‐based Jupyter notebook implementation of MassQL. Whereas MassQL provides the language to apply one query to one file, MassQLab uses MassQL to apply a series of queries to a series of files and tabulates and visualizes the results. MassQLab was developed using Python 3.9 and is made publicly available (https://github.com/JohnsonDylan/MassQLab). The software environment utilizes JupyterLab for interactive computing, MassQL for querying mass spectrometry data, and matplotlib for visualizations. Tabular outputs are formatted using openpyxl, and reports are generated via reportlab. A standalone executable version of MassQLab is being developed for future release.

A MassQL query operates using a structured query language (SQL) but with terms familiar to mass spectrometrists (Table S1). Users should refer to MassQL documentation for specific instruction on composing queries and reusing queries shared via the MassQL compendium [8].

MassQLab uses a configuration file that defines (1) the directory where mzML files to be processed are stored (mzML directory) and (2) the filepath of the query file (.xlsx or .json) that contains the arguments that compose each named MassQL query. MassQLab applies each query in the query file to each mzML file in the mzML directory. The area of the peak (if present) for each query run is calculated and tabulated for export as an .xlsx file. The results include several graphical outputs for quick review as well as tabulated data. A resultant PDF file is also generated that contains plots of the results for each named MassQL query. Users should refer to the package documentation for detailed instruction on MassQLab operation.

## 3 Results and Discussion

### 3.1 MassQLab Facilitates SST

MassQLab removes barriers to SST implementation by empowering users to select standards, mass spectrometer, hyphenated analysis techniques (e.g., LC or GC), and data acquisition method. The only prerequisites are MS data in mzML open‐source format and a MassQL query (created or reused). As an illustrative use case, we utilized MassQLab in the development of an SST protocol in our laboratory. Given the nature of our research, it was desirable to use a set of standards mirroring our focus on endogenous metabolite measurement. We, therefore, chose a set of deuterated acylcarnitines (Table 1) as they were commercially available, not expected in biological samples, and span the chromatographic and mass range of our LC–MS method.

**TABLE 1:** Tabulated information and MassQL queries for deuterated acylcarnitines.

Chemical	Molecular formula	*m/z*: [M + H]^+^	RT (minutes): min, max	Product ions (*m/z*)
**Acetyl‐L‐carnitine‐d**_**3**_	C_9_H_14_D_3_NO_4_	207.1419	1.11, 1.44	148.0684, 85.0284
**Propionyl‐L‐carnitine‐d**_**3**_	C_10_H_16_D_3_NO_4_	221.1575	3.28, 3.61	159.0650, 85.0284
**Valeryl‐L‐carnitine‐d**_**3**_	C_12_H_20_D_3_NO_4_	249.1888	4.13, 4.46	190.1154, 85.0284
**Octanoyl‐L‐carnitine‐d**_**3**_	C_15_H_26_D_3_NO_4_	291.2358	5.74, 6.07	229.1435, 85.0284
**Lauroyl‐L‐carnitine‐d**_**3**_	C_19_H_34_D_3_NO_4_	347.2984	7.44, 7.77	183.1744, 85.0284
**Oleoyl‐L‐carnitine‐d**_**3**_	C_25_H_44_D_3_NO_4_	429.3766	9.27, 9.59	367.2804, 85.0284

The conventional approach to assessing the SST mix (deuterated acylcarnitines) was to manually assess raw LC–MS files (.raw) using vendor‐supported software (Freestyle, Thermo). One would calculate the monoisotopic *m/z* for each ion in the SST and generate an extracted ion chromatogram (EIC). The retention time (RT), peak shape, and other metrics of chromatographic performance can be evaluated subjectively (e.g., overlay of prior data) or objectively by comparing measured values with historical values in a spreadsheet. The mass spectrum, typically at the apex of the chromatographic elution, of each chemical can likewise be interpreted for the mass accuracy, isotope pattern (if applicable), and other mass‐to‐charge values (*m/z*) present in the spectrum (e.g., in‐source fragments). If tandem mass spectrometry (MS/MS or MS2) is performed, that information, such as an MS/MS product ion spectrum, can be manually reviewed and compared with prior data. The MassQLab workflow reduces many of these manual steps and evaluations.

**FIGURE 1:**
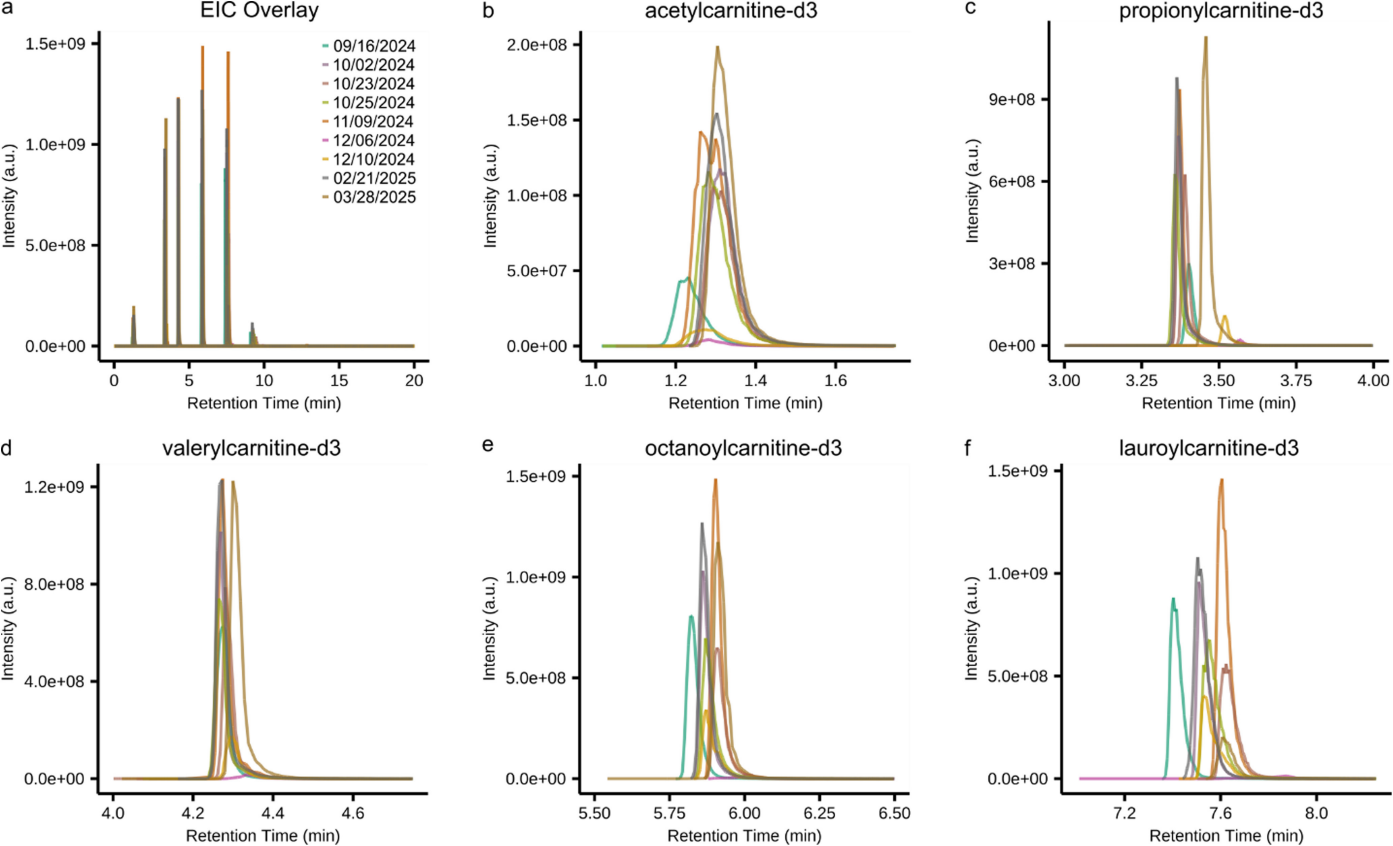
MassQLab assessment of retention time in SST. (a) Overlay of extracted ion chromatogram (EIC) of deuterated acylcarnitines in SST mixture and zoomed retention time displaying (b) acetyl‐L‐carnitine‐d3, (c) propionyl‐L‐carnitine‐d3, (d) valeryl‐L‐carnitine‐d3, (e) octanoyl‐L‐carnitine‐d3, and (f) lauroyl‐L‐carnitine‐d3. Line color indicates SST mixture analysis date.

SST mixture data were acquired over the course of approximately 6 months on two different instrument platforms (Vanquish Horizon—Orbitrap Fusion and Vanquish Horizon—Orbitrap Fusion Lumos) running identical LC–MS methods, adapting the instrument configuration (e.g., tubing length) to match differences in flow path, diameters, and materials (assessed by elution time of the SST mixture). These data were acquired by five different expert‐level mass spectrometrists prior to the analysis of a variety of matrices and experimental designs. Data were processed using MassQLab and the MassQL queries listed in Table S1. MassQLab optionally provides numerous outputs including EICs, integrated peak areas, and comparisons between multiple files. The MassQLab output was replotted to generate the overlaid EICs displayed in Figure 1a. The individual query results are displayed in their respective chromatographic elution time window in Figure 1b–f, Figure S1.

To demonstrate the construction of the MassQL query, we detail the steps for valeryl‐L‐carnitine‐d_3_ (Figure 1d). The analyst must determine the ionization mode, adduct, and corresponding monoisotopic mass‐to‐charge (*m/z*) of the desired precursor. There is no requirement that the protonated (or deprotonated) adduct be used, and thus, the analyst is fully empowered to use the most suitable *m/z* based on their circumstances. In the case of valeryl‐L‐carnitine‐d_3_, a zwitterionic acylcarnitine chemical, the protonated adduct was determined to be ideal with a precursor value of *m/z* 249.1888, indicated in the MassQL query by MS1MZ = 249.1888. Next, the tolerance of the *m/z* value should be indicated via the MassQL query via either a relative (TOLERANCEPPM = 10) or absolute value (TOLERANCEMZ). The query can be further refined to include isotope patterns (e.g., ^13^C or ^34^S), concurrently detected *m/z* values from multiple adducts (e.g., [M + H]^+^ and [M + Na]^+^), relative intensity, or other mass spectral characteristics unique to the SST analytes. Additional terms increase the specificity but can be detrimental to MassQLab processing speed. Inclusion of chromatographic elution is performed by defining a retention time minimum (RTMIN = 4.13), maximum (RTMAX = 4.46), or window (RTMIN = 4.13 AND RTMAX = 4.46). Analogously, values obtained by ion mobility spectrometry can be defined to provide additional specificity to the MassQL query.

Regarding the SST evaluation in our illustrative data herein, we noted the visual shifting in retention times, most notably in the cases of octanoyl‐L‐carnitine‐d_3_, lauroyl‐L‐carnitine‐d_3_, and oleoyl‐L‐carnitine‐d_3_. Statistical assessment of retention time, Table S2, yielded a relative standard deviation (RSD) in all SST analytes ranging from 0.5% to 2.3%. This is visually reflected in the barbell plot displayed in Figure S2a. We found this value to be acceptable given the number of variables that were not controlled for in this illustrative case, such as different personnel, different column and guard column, and different LC solvents. Note, the intensity and peak shape were not evaluated. Additionally, a result obtained in the MassQLab output implicitly assesses mass accuracy. Outputs are not returned from MassQLab if measured beyond the tolerance specified in the MassQL query, which assesses *m/z* of individual scans and is not reliant on centroid or average *m/z* values.

In the course of this work, we discovered that the components of the SST mixture degraded over time, namely, oleoyl‐L‐carnitine‐d_3_, and adversely affected the abundance comparisons. This is visually noted in the EIC (Figure S1) and in the plotting of the mean peak area (Figure S2b). The observed, seemingly selective degradation of the oleoyl‐L‐carnitine‐d_3_ analyte was counterintuitive. All acylcarnitine SST analytes share similar structural features, are exposed to the same experimental conditions, and are equally susceptible to nonenzymatic hydrolysis. While we could not explain the rationale for this observation, we effectively counteracted this phenomenon by implementing stricter and consistent SST mixture storage conditions and time‐use limitations (used for no more than 1 week stored at 4°C).

### 3.2 MassQLab Assessment of MS/MS Spectral Consistency

One important, and not often assessed, aspect of SST is MS/MS consistency. This is particularly important when using MS/MS for feature annotation in untargeted mass spectrometry experiments, e.g., metabolomics. As a proof‐of‐concept, we used MassQLab to evaluate MS/MS data obtained using DDA. We analyzed our SST mixture over a 5‐day period, once daily, on an UHPLC‐HRMS platform (Vanquish Horizon—Orbitrap Fusion) using multiple collision energies as well as resonant and nonresonant excitation methods, CID and HCD, respectively. The DDA was scheduled using a precursor *m/z* inclusion list at which point a survey MS1 scan would be collected followed immediately by a series of sequential HCD scans at increasing NCE followed by CID scans at increasing NCE.

**FIGURE 2:**
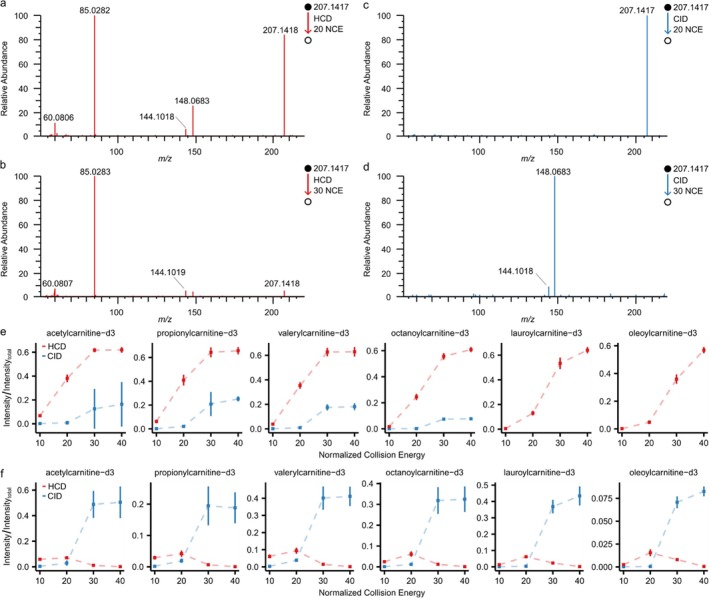
MassQLab assessment of MS/MS consistency as part of SST. Illustrative MS/MS product ion spectrum for observed *m/z* 207.1417 (acetyl‐L‐carnitine‐d_3_) obtained by (a) HCD at 20 NCE, (b) HCD at 30 NCE, (c) CID at 20 NCE, and (d) CID at 30 NCE. Plots displaying the mean and standard deviation of the normalized intensity of (e) *m/z* 85.0284 product ion or (f) neutral losses of trimethylamine (or trimethylamine‐d3) acquired using HCD or CID at different normalized collision energy (NCE) from SST mixture components over five consecutive days of analysis.

Acylcarnitines have consistent fragmentation patterns resulting from common substructures [9], a common product ion of *m/z* 85.0284 and neutral loss of trimethylamine (or deuterated analog). The generation of the anticipated product ions and neutral losses is energy dependent and anticipated to change between CID and HCD, Figure 2a–d. The location of the deuteration alters the anticipated SST analyte queries; deuteration was located on the trimethylamine substructure or on the acyl fatty acid chain. We created MassQL queries for the six deuterated acylcarnitines in the SST mixture, Table 1; specifically, we chose to write queries for the anticipated product ions, but one could use neutral loss queries, equivalently.

The results of analyzing the 5‐day DDA data using MassQLab are plotted in Figure 2. To ensure comparable scaling across samples, we normalized the product ion intensity to the intensity of the product ion scan. This accounts for the variance in MS/MS product ion spectra acquisition, and corresponding total signal, during chromatographic elution. The mean and standard deviation were computed and plotted in Figure 2e. Consistent trends were observed. The *m/z* 85.0824 signal increased logarithmically for CID and HCD. CID produced a lower signal for *m/z* 85.0824 compared to HCD at all normalized collision energies (NCE) tested. Contrastingly, the observation of product ions originating from the neutral loss of trimethylamine (or the deuterated analog) was differential between CID and HCD (Figure 2f). CID at NCE above 30 yielded more signal, whereas HCD yielded nearly undetectable levels of signal. Our data suggest that, for identification purposes, generating two characteristic fragment ions via CID at NCE > 30 is superior for this set of deuterated acylcarnitines. In contrast, quantification of the product ion *m/z* 85.0824 may be facilitated by HCD.

### 3.3 MassQLab Reveals SST Deficiencies

**FIGURE 3:**
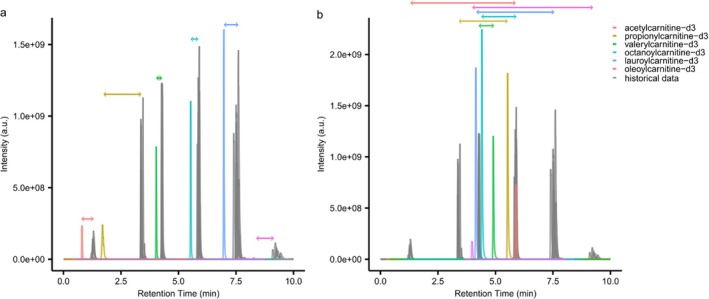
MassQLab facilitated detection of system deficiencies. (a) nonlinear shifts in retention time in SST (color traces by analyte) compared to anticipated values from 6‐month data due to column lot‐to‐lot variation. (b) incorrect column and chromatography method (color traces by analyte) compared to anticipated values yielded notable differences in elution order and time. Double‐headed arrows connect the measured to anticipated elution time by analyte indicated by color.

Deficiencies in system performance and user errors, an inevitability, are readily detected with MassQLab outputs as illustrated in Figure 3a,b, replotted to improve visualization. In Figure 3a, SST and MassQLab aided in the recognition of retention time shifts (colored traces compared to gray), later attributed to a column‐specific issue of unknown origin putatively linked to a specific lot of columns. This atypical performance was noted under identical chromatography solvents, equipment, and conditions compared to other columns from different lots. In a second example (Figure 3b), the MassQLab output, specifically the analyte EICs, was erroneous. The SST mixture elution times were almost in reverse order. Upon review of the system, a BEH Amide column and incorrect chromatographic method were used, left over from the prior day's experiment. Using our SST strategy empowered by MassQLab, we successfully recognized and were able to correct performance issues prior to initiating full experimental analysis.

## 4 Conclusion

SST is crucial for obtaining high‐quality mass spectrometry data. In the development of MassQLab, we aimed to reduce barriers to SST implementation by empowering users to select the standards required for their specific use cases, reuse MassQL query information, and broad compatibility (vendor and configuration). This is accomplished by removing the inefficiencies of learning multiple vendor‐specific software, replacing it with a universal and flexible language, MassQL, which can be used on any mass spectrometry platform. We demonstrate the operation and function of MassQLab and discuss observations learned during its implementation. Importantly, MassQLab possesses the basic functions required of an MS software tool, such as EIC generation. Further, we assessed MS/MS data acquired over multiple days. Our data indicate reproducibility in the product ions and their relative intensity, and that SST MS/MS evaluation is not warranted in our use case. One rationale for this conclusion is that the stochastic nature of MS/MS triggering using DDA is problematic for assessing consistency using SST, albeit normalization to the total signal helped in our evaluation. More impactfully, DDA limited the number of MS1 scans obtained and, therefore, negatively impacted the interpretation of retention time and similar characteristics (viz., peak shape). We demonstrated how historical data can be assessed to retrospectively discover patterns, but more importantly be used to identify instrument performance issues prior to data collection. In future directions, we plan to refine the performance of MassQLab and expand upon the functions available to users.

## Author Contributions

**Heather L. Winter:** validation, writing – review and editing, formal analysis, writing – original draft, investigation, methodology, conceptualization, visualization. **Dylan Johnson:** software, validation, visualization, investigation, writing – original draft, writing – review and editing, conceptualization. **Alan K. Jarmusch:** conceptualization, investigation, writing – review and editing, validation, visualization, project administration, supervision, resources.

## Supporting information


**Table S1:** Tabulated MassQL queries.
**Figure S1:** Overlay of extracted ion chromatograms of oleoy‐L‐carnitine‐d_3_ collected between Sept 2024 to March 2025.
**Table S2:** Tabulated retention time metrics from SST evaluation spanning Sept 2024 to March 2025.
**Figure S2:** Historical SST data over 6 months. (a) Barbell plot reflecting retention time with the center (black) point indicated mean and left (blue) and right (red) points indicated the standard deviation. (b) Bar plot indicating the mean peak area with standard deviation error bars.

## Data Availability

The data that support the findings of this study are openly available in MassIVE at https://massive.ucsd.edu/ProteoSAFe/dataset.jsp?accession=MSV000098825, reference number MSV000098825.
