# Supplementary material for: Open‐Source System Suitability: Mass Spectrometry Query Language Lab (MassQLab)
Source: Rapid Commun Mass Spectrom. 2025 Sep 2;39(23):e10132. doi: 10.1002/rcm.10132 (PMC12404904; doi:10.1002/rcm.10132)
Supplement: Supplementary file 1 — Table S1: Tabulated MassQL queries. Figure S1: Overlay of extracted ion chromatograms of oleoy‐L‐carnitine‐d3 collected between Sept 2024 to March 2025. Table S2: Tabulated retention time metrics from SST evaluation spanning Sept 2024 to March 2025. Figure S2: Historical SST data over 6 months. (a) Barbell plot reflecting retention time with the center (black) point indicated mean and left (blue) and right (red) points indicated the standard deviation. (b) Bar plot indicating the mean peak area with standard deviation error bars. [file RCM-39-e10132-s001.docx]

## Supplementary Information

Open-source System Suitability: Mass Spectrometry Query Language Lab (MassQLab)

Heather L. Winter,^1†^ Dylan Johnson,^2†^ and Alan K. Jarmusch^1*^

^1^ Metabolomics Core Facility, Immunity, Inflammation, and Disease Laboratory, Division of Intramural Research, National Institute of Environmental Health Sciences, National Institutes of Health, Research Triangle Park, NC 27709, USA

^2^ Integrative Bioinformatics, National Institute of Environmental Health Sciences, National Institutes of Health, Research Triangle Park, NC 27709, USA

† Equal contribution

* Corresponding author

# Supplementary Figures and Tables

**Table S1. Tabulated MassQL Queries**

| Chemical | MS Level | Query | Rationale |
| --- | --- | --- | --- |
| acetylcarnitine-d_3_ | MS | QUERY scaninfo(MS1DATA) FILTER MS1MZ=207.1419:TOLERANCEPPM=10 AND RTMIN=1.03 AND RTMAX=1.36 | Assessment of MS1 data matching precursor *m/z* within mass tolerance (ppm) between specified retention time (minutes). |
|  | MS/MS | QUERY scaninfo(MS2DATA) WHERE MS2PREC=207.1419:TOLERANCEPPM=10 AND RTMIN=1.03 AND RTMAX=1.36 FILTER MS2PROD=85.0284:TOLERANCEPPM=10 | Assessment of MS2 data matching precursor *m/z* within mass tolerance (ppm) between specified retention time (minutes) and return intensity for the MS2 product ion within mass tolerance (ppm). |
|  | MS/MS | QUERY scaninfo(MS2DATA) WHERE MS2PREC=207.1419:TOLERANCEPPM=10 AND RTMIN=1.03 AND RTMAX=1.36 FILTER MS2PROD=148.0684:TOLERANCEPPM=10 | Assessment of MS2 data matching precursor *m/z* within mass tolerance (ppm) between specified retention time (minutes) and return intensity for the MS2 product ion within mass tolerance (ppm). |
|  | MS/MS | QUERY scaninfo(MS2DATA) WHERE MS2PREC=207.1419:TOLERANCEPPM=10 AND RTMIN=1.03 AND RTMAX=1.36 | Assessment of MS2 data matching precursor *m/z* within mass tolerance (ppm) between specified retention time (minutes) and return intensity for all signals in the MS2 spectrum. |
| propionylcarnitine-d_3_ | MS | QUERY scaninfo(MS1DATA) FILTER MS1MZ=221.1575:TOLERANCEPPM=10 AND RTMIN=3.21 AND RTMAX=3.54 | Assessment of MS1 data matching precursor *m/z* within mass tolerance (ppm) between specified retention time (minutes). |
|  | MS/MS | QUERY scaninfo(MS2DATA) WHERE MS2PREC=221.1575:TOLERANCEPPM=10 AND RTMIN=3.21 AND RTMAX=3.54 FILTER MS2PROD=85.0284:TOLERANCEPPM=10 | Assessment of MS2 data matching precursor *m/z* within mass tolerance (ppm) between specified retention time (minutes) and return intensity for the MS2 product ion within mass tolerance (ppm). |
|  | MS/MS | QUERY scaninfo(MS2DATA) WHERE MS2PREC=221.1575:TOLERANCEPPM=10 AND RTMIN=3.21 AND RTMAX=3.54 FILTER MS2PROD=159.0626:TOLERANCEPPM=10 | Assessment of MS2 data matching precursor *m/z* within mass tolerance (ppm) between specified retention time (minutes) and return intensity for the MS2 product ion within mass tolerance (ppm). |
|  | MS/MS | QUERY scaninfo(MS2DATA) WHERE MS2PREC=221.1575:TOLERANCEPPM=10 AND RTMIN=3.21 AND RTMAX=3.54 | Assessment of MS2 data matching precursor *m/z* within mass tolerance (ppm) between specified retention time (minutes) and return intensity for all signals in the MS2 spectrum. |
| valerylcarnitine-d_3_ | MS | QUERY scaninfo(MS1DATA) FILTER MS1MZ=249.1888:TOLERANCEPPM=10 AND RTMIN=4.07 AND RTMAX=4.40 | Assessment of MS1 data matching precursor *m/z* within mass tolerance (ppm) between specified retention time (minutes). |
|  | MS/MS | QUERY scaninfo(MS2DATA) WHERE MS2PREC=249.1888:TOLERANCEPPM=10 AND RTMIN=4.07 AND RTMAX=4.40 FILTER MS2PROD=85.0284:TOLERANCEPPM=10 | Assessment of MS2 data matching precursor *m/z* within mass tolerance (ppm) between specified retention time (minutes) and return intensity for the MS2 product ion within mass tolerance (ppm). |
|  | MS/MS | QUERY scaninfo(MS2DATA) WHERE MS2PREC=249.1888:TOLERANCEPPM=10 AND RTMIN=4.07 AND RTMAX=4.40 FILTER MS2PROD=190.1154:TOLERANCEPPM=10 | Assessment of MS2 data matching precursor *m/z* within mass tolerance (ppm) between specified retention time (minutes) and return intensity for the MS2 product ion within mass tolerance (ppm). |
|  | MS/MS | QUERY scaninfo(MS2DATA) WHERE MS2PREC=249.1888:TOLERANCEPPM=10 AND RTMIN=4.07 AND RTMAX=4.40 | Assessment of MS2 data matching precursor *m/z* within mass tolerance (ppm) between specified retention time (minutes) and return intensity for all signals in the MS2 spectrum. |
| octanoylcarnitine-d_3_ | MS | QUERY scaninfo(MS1DATA) FILTER MS1MZ=291.2358:TOLERANCEPPM=10 AND RTMIN=5.64 AND RTMAX=5.98 | Assessment of MS1 data matching precursor *m/z* within mass tolerance (ppm) between specified retention time (minutes). |
|  | MS/MS | QUERY scaninfo(MS2DATA) WHERE MS2PREC=291.2358:TOLERANCEPPM=10 AND RTMIN=5.64 AND RTMAX=5.98 FILTER MS2PROD=85.0284:TOLERANCEPPM=10 | Assessment of MS2 data matching precursor *m/z* within mass tolerance (ppm) between specified retention time (minutes) and return intensity for the MS2 product ion within mass tolerance (ppm). |
|  | MS/MS | QUERY scaninfo(MS2DATA) WHERE MS2PREC=291.2358:TOLERANCEPPM=10 AND RTMIN=5.64 AND RTMAX=5.98 FILTER MS2PROD=229.1409:TOLERANCEPPM=10 | Assessment of MS2 data matching precursor *m/z* within mass tolerance (ppm) between specified retention time (minutes) and return intensity for the MS2 product ion within mass tolerance (ppm). |
|  | MS/MS | QUERY scaninfo(MS2DATA) WHERE MS2PREC=291.2358:TOLERANCEPPM=10 AND RTMIN=5.64 AND RTMAX=5.98 | Assessment of MS2 data matching precursor *m/z* within mass tolerance (ppm) between specified retention time (minutes) and return intensity for all signals in the MS2 spectrum. |
| lauroylcarnitine-d_3_ | MS | QUERY scaninfo(MS1DATA) FILTER MS1MZ=347.2984:TOLERANCEPPM=10 AND RTMIN=7.27 AND RTMAX=7.61 | Assessment of MS1 data matching precursor *m/z* within mass tolerance (ppm) between specified retention time (minutes). |
|  | MS/MS | QUERY scaninfo(MS2DATA) WHERE MS2PREC=347.2984:TOLERANCEPPM=10 AND RTMIN=7.27 AND RTMAX=7.61 FILTER MS2PROD=85.0284:TOLERANCEPPM=10 | Assessment of MS2 data matching precursor *m/z* within mass tolerance (ppm) between specified retention time (minutes) and return intensity for the MS2 product ion within mass tolerance (ppm). |
|  | MS/MS | QUERY scaninfo(MS2DATA) WHERE MS2PREC=347.2984:TOLERANCEPPM=10 AND RTMIN=7.27 AND RTMAX=7.61 FILTER MS2PROD=285.2035:TOLERANCEPPM=10 | Assessment of MS2 data matching precursor *m/z* within mass tolerance (ppm) between specified retention time (minutes) and return intensity for the MS2 product ion within mass tolerance (ppm). |
|  | MS/MS | QUERY scaninfo(MS2DATA) WHERE MS2PREC=347.2984:TOLERANCEPPM=10 AND RTMIN=7.27 AND RTMAX=7.61 | Assessment of MS2 data matching precursor *m/z* within mass tolerance (ppm) between specified retention time (minutes) and return intensity for all signals in the MS2 spectrum. |
| oleoylcarnitine-d_3_ | MS | QUERY scaninfo(MS1DATA) FILTER MS1MZ= 429.3766:TOLERANCEPPM=10 AND RTMIN=8.95 AND RTMAX=9.29 | Assessment of MS1 data matching precursor *m/z* within mass tolerance (ppm) between specified retention time (minutes). |
|  | MS/MS | QUERY scaninfo(MS2DATA) WHERE MS2PREC=429.3766:TOLERANCEPPM=10 AND RTMIN=8.95 AND RTMAX=9.29 FILTER MS2PROD=85.0284:TOLERANCEPPM=10 | Assessment of MS2 data matching precursor *m/z* within mass tolerance (ppm) between specified retention time (minutes) and return intensity for the MS2 product ion within mass tolerance (ppm). |
|  | MS/MS | QUERY scaninfo(MS2DATA) WHERE MS2PREC=429.3766:TOLERANCEPPM=10 AND RTMIN=8.95 AND RTMAX=9.29 FILTER MS2PROD=367.2817:TOLERANCEPPM=10 | Assessment of MS2 data matching precursor *m/z* within mass tolerance (ppm) between specified retention time (minutes) and return intensity for the MS2 product ion within mass tolerance (ppm). |
|  | MS/MS | QUERY scaninfo(MS2DATA) WHERE MS2PREC=429.3766:TOLERANCEPPM=10 AND RTMIN=8.95 AND RTMAX=9.29 | Assessment of MS2 data matching precursor *m/z* within mass tolerance (ppm) between specified retention time (minutes) and return intensity for all signals in the MS2 spectrum. |


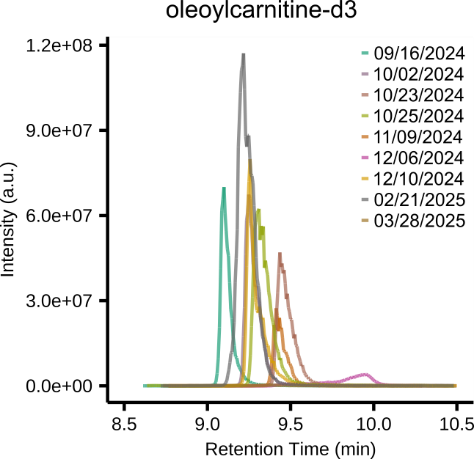


**Figure S1**. Overlay of extracted ion chromatograms of oleoylcarnitine-d_3_ collected between Sept 2024 to March 2025.

**Table S2.** Tabulated retention time metrics from SST evaluation spanning Sept 2024 to March 2025.

|  | **Vanquish-Fusion RT (min)** | | | **Vanquish-Lumos RT (min)** | | | **Combined RT (min)** | | |
| --- | --- | --- | --- | --- | --- | --- | --- | --- | --- |
| **Chemical** | **Mean** | **SD** | **RSD (%)** | **Mean** | **SD** | **RSD (%)** | **Mean** | **SD** | **RSD (%)** |
| acetylcarnitine-d_3_ | 1.27 | 0.03 | 2.4 | 1.31 | 0.01 | 0.8 | 1.29 | 0.03 | 2.3 |
| propionylcarnitine-d_3_ | 3.47 | 0.09 | 2.6 | 3.39 | 0.04 | 1.2 | 3.42 | 0.08 | 2.3 |
| valerylcarnitine-d_3_ | 4.30 | 0.03 | 0.7 | 4.28 | 0.02 | 0.5 | 4.29 | 0.02 | 0.5 |
| octanoylcarnitine-d_3_ | 5.90 | 0.08 | 1.4 | 5.89 | 0.03 | 0.5 | 5.89 | 0.05 | 0.8 |
| lauroylcarnitine-d_3_ | 7.61 | 0.19 | 2.5 | 7.57 | 0.05 | 0.7 | 7.59 | 0.12 | 1.6 |
| oleoylcarnitine-d_3_ | 9.26 | 0.16 | 1.7 | 9.33 | 0.09 | 1.0 | 9.30 | 0.11 | 1.2 |


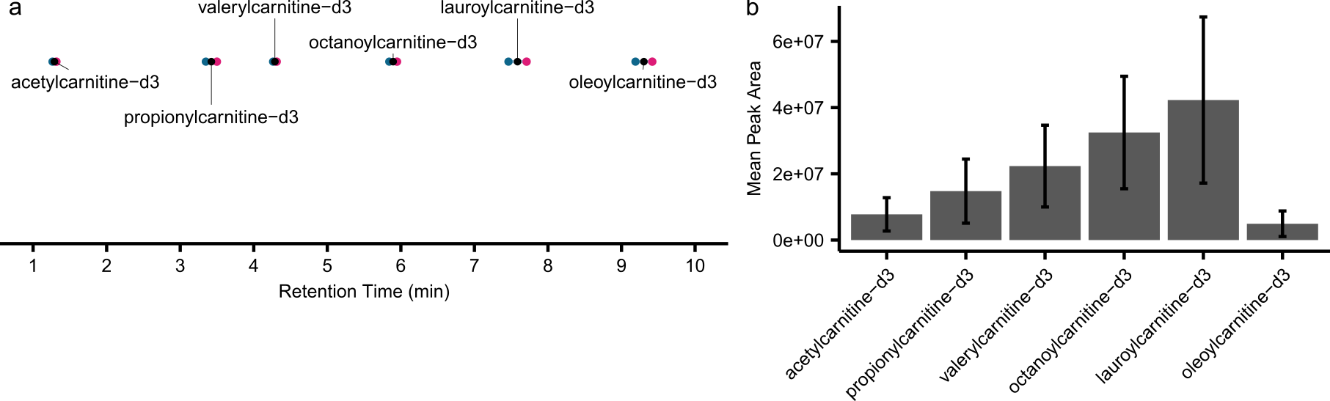
 **Figure S2**. Historical SST data over 6 months. (a) Barbell plot reflecting retention time with the center (black) point indicated mean and left (blue) and right (red) points indicated the standard deviation. (b) Bar plot indicating the mean peak area with standard deviation error bars.
